# Genome-Wide Association Study of Seed Dormancy and the Genomic Consequences of Improvement Footprints in Rice (*Oryza sativa* L.)

**DOI:** 10.3389/fpls.2017.02213

**Published:** 2018-01-05

**Authors:** Qing Lu, Xiaojun Niu, Mengchen Zhang, Caihong Wang, Qun Xu, Yue Feng, Yaolong Yang, Shan Wang, Xiaoping Yuan, Hanyong Yu, Yiping Wang, Xiaoping Chen, Xuanqiang Liang, Xinghua Wei

**Affiliations:** ^1^State Key Laboratory of Rice Biology, China National Rice Research Institute, Hangzhou, China; ^2^Crops Research Institute, Guangdong Academy of Agricultural Sciences, South China Peanut Sub-Center of National Center of Oilseed Crops Improvement and Guangdong Provincial Key Laboratory of Crop Genetic Improvement, Guangzhou, China

**Keywords:** genome-wide association study, seed dormancy, selective sweeps, improvement footprints, rice (*Oryza sativa* L.)

## Abstract

Seed dormancy is an important agronomic trait affecting grain yield and quality because of pre-harvest germination and is influenced by both environmental and genetic factors. However, our knowledge of the factors controlling seed dormancy remains limited. To better reveal the molecular mechanism underlying this trait, a genome-wide association study was conducted in an *indica*-only population consisting of 453 accessions genotyped using 5,291 SNPs. Nine known and new significant SNPs were identified on eight chromosomes. These lead SNPs explained 34.9% of the phenotypic variation, and four of them were designed as dCAPS markers in the hope of accelerating molecular breeding. Moreover, a total of 212 candidate genes was predicted and eight candidate genes showed plant tissue-specific expression in expression profile data from different public bioinformatics databases. In particular, LOC_Os03g10110, which had a maize homolog involved in embryo development, was identified as a candidate regulator for further biological function investigations. Additionally, a polymorphism information content ratio method was used to screen improvement footprints and 27 selective sweeps were identified, most of which harbored domestication-related genes. Further studies suggested that three significant SNPs were adjacent to the candidate selection signals, supporting the accuracy of our genome-wide association study (GWAS) results. These findings show that genome-wide screening for selective sweeps can be used to identify new improvement-related DNA regions, although the phenotypes are unknown. This study enhances our knowledge of the genetic variation in seed dormancy, and the new dormancy-associated SNPs will provide real benefits in molecular breeding.

## Introduction

Plant seeds, especially cereal grains, are vitally important sources of human nutrition, providing half of the global per capita energy intake. Consequently, various seed traits such as seed dormancy, which is regarded as the failure of an intact viable seed to complete germination under favorable conditions (Bewley, 1997), have been under strong artificial and natural selection during crop domestication (Kovach et al., 2007; Izawa et al., 2009). Because seed dormancy is closely related to pre-harvest sprouting (PHS), it is one of the most important traits in rice breeding programs (Bewley and Black, 1982). Rice seed dormancy is like a double-edged sword in terms of cultivation and utilization. Weak dormancy leads to a higher PHS rate in rainy weather and results in production losses and poor quality. In southern China, because of the long rainy season, it causes heavy PHS of 5–20% of hybrid rice grains (Hu et al., 2003). However, deep seed dormancy, especially for hybrid seeds, can cause non-uniform germination or even prevent germination in the process of sowing. Thus, balancing the advantages and disadvantages of seed dormancy is an important goal in rice breeding. Moreover, understanding the genetic variation of seed dormancy is of great interest to plant breeders.

The expression of seed dormancy is influenced by environmental factors such as temperature and humidity (Basbouss-Serhal et al., 2016), endogenous hormones such as abscisic acid (ABA), gibberellic acid (GA), and the ABA-to-GA ratio (White and Rivin, 2000; Fang et al., 2008; Zhang H. et al., 2009; Gu et al., 2011; Ye et al., 2015) and some special biological tissues such as the embryo, endosperm and maternal tissues (e.g., the seed coat) (Gu et al., 2015). Recently, many quantitative trait loci (QTLs) affecting seed dormancy or germination-related traits have been identified in plant species such as barley (Ullrich et al., 1993; Gao et al., 2003; Li et al., 2004), wheat (Flintham et al., 2002; Kulwal et al., 2010; Liu et al., 2013), rice (Cai and Morishima, 2000; Guo et al., 2004; Wan et al., 2006; Gu et al., 2010; Sugimoto et al., 2010; Xie et al., 2011; Lee et al., 2017), oats (Fennimore et al., 1999), sorghum (Lijavetzky et al., 2000), Arabidopsis (Alonso-Blanco et al., 2003; Amiguet-Vercher et al., 2015), sunflower (Gandhi et al., 2005), rye (Masojć et al., 2007, 2009), rapeseed (Feng et al., 2009; Schatzki et al., 2013), and lettuce (Huo et al., 2013). However, only a few QTLs have been finely mapped and cloned in rice, such as *qSD12* (Gu et al., 2010) on chromosome 12, *Sdr4* (Sugimoto et al., 2010) and *qSD7-1/Rc* (Gu et al., 2011) on chromosome 7 and *qSD1-2* (Ye et al., 2015) on chromosome 1.

Plant seed dormancy is a complex agronomic trait. Traditional bi-parental QTL mapping is limited by the recombination events occurring over a few generations during the development of a recombination inbreed line population. Therefore, most of the biological mechanisms of seed dormancy have not been clearly elucidated. In previous reports, only three genes, *qSD12* (Gu et al., 2010), *qSD7-1* (Gu et al., 2011), and *qSD1-2* (Ye et al., 2015), were identified to regulate hormone accumulation in developing or mature seeds in rice. Thus, more novel QTLs for this trait need to be isolated by highly efficient and reliable QTL mapping methods in the future. Genome-wide association studies (GWASs) based on the historic recombination in a large natural population, a high-density SNP map and a comprehensive HapMap have become a powerful complementary approach for linkage mapping to identify complex trait variation at the genome-wide level (Huang et al., 2010). GWASs can overcome the limitations of traditional bi-parental populations and dissect complex traits with high mapping resolution. In the past few years, GWASs have been successfully applied in the dissection of complex traits in humans (Edwards et al., 2005), animals (Duijvesteijn et al., 2010), and plant species such as *Arabidopsis thaliana* (Atwell et al., 2010), soybean (Wen et al., 2014; Zhang et al., 2015), wheat (Sukumaran et al., 2015), barley (Gawenda et al., 2015), maize (Zhang et al., 2014), sorghum (Zhang D. et al., 2015), tomato (Zhang et al., 2016a), rapeseed (Qu et al., 2015), sesame (Wei et al., 2015), and *Aegilops tauschii* (Liu et al., 2015). In rice, GWASs have also been applied widely to detect novel QTLs involved in different complex traits, for example yield (Begum et al., 2015; Huang X. et al., 2015), environmental stress resistance (Kumar et al., 2015; Lv et al., 2015), grain quality (Huang Y. et al., 2015), blast resistance (Wang C. et al., 2014; Wang et al., 2015), flowering time (Huang et al., 2011) and also seed dormancy (Magwa et al., 2016). Nowadays, together with linkage analysis, the GWAS strategy is playing an increasingly important role in dissecting novel QTLs and uncovering whole genome-wide variation.

Rice domestication has been a hot research topic for a long time. Many domestication related-genes have been mapped or cloned in previous studies, such as *PROG1* (Jin et al., 2008; Tan et al., 2008), *An1* (Luo et al., 2013), *sh4* (Li et al., 2006), *Bh4* (Zhu et al., 2011), and *sd1* (Asano et al., 2011). Seed dormancy is a typical and direct domestication-related trait that has been strongly affected by nature and human selection over the long history of rice domestication. Two seed dormancy genes, *Sdr4* (Sugimoto et al., 2010) and *qSD7-1/Rc* (Gu et al., 2011), have been proven to be directly involved in rice domestication. Long-term domestication has dramatically changed phenotypes, reduced allele frequencies, genetic diversity and polymorphism information content (PIC), and drastically increased linkage disequilibrium (LD) (Richards et al., 2004; Chen et al., 2010; Qanbari et al., 2014). Many methods based on the high density of single nucleotide polymorphism (SNP) markers have been used to detect genomic selection signals, such as nucleotide diversity (π) (Huang et al., 2012), PIC (Rostoks et al., 2006), population differentiation (*F*_ST_) (Wilkinson et al., 2013), cross-population composite likelihood ratio (XP-CLR) (Xu et al., 2011) and extended haplotype homozygosity (EHH) (Sabeti et al., 2002; Olsen et al., 2006). In the case of rice, multiple selective sweeps have been detected using these methods (Olsen et al., 2006; Huang et al., 2012; Huang X. et al., 2015). Therefore, selective sweep analysis is another approach to identify QTLs for domestication traits.

In this study, on the basis of our previous reports (Lu et al., 2015), a high-density custom- designed array containing 5,291 SNPs was used to genotype 453 *indica* accessions. The aims of our research were (1) to identify a substantial number of significantly associated SNPs and some putative genes potentially regulating seed dormancy in the whole panel; (2) to design dCAPS markers for different alleles of the associated SNPs for breeding applications; (3) to analyze the genetic diversity population structure and LD of the landraces and improved lines; and (4) to detect selective sweep regions using PIC ratio statistics between the landraces and improved lines.

## Materials and methods

### Plant materials and SNP genotypes

The association mapping population consisted of 453 *indica* accessions previously described in detail by Lu et al. (2015). According to the pedigree information, these accessions were classified into three types: landraces (266), improved lines (89) and foreign introduced lines (81) (Table S1). A rice landrace was defined as a “farm cultivar”, “traditional variety,” or “local variety” that had developed over a long time and adapted to the local natural and cultural environment. Rice landraces have always maintained rich genetic diversity. An improved line was defined as a “breeding variety” that was artificially selected by a breeder to meet a specific economic need, such as high yield, fine quality or multi-resistance. The genetic diversity of the improved lines was lower than that of the landraces. Therefore, the PIC ratios of the landraces (266) and improved lines (89) were used to detect whole-genome selective sweep signals. All accessions were planted in a randomized complete block design with three field replications and each line was planted in six rows with six hills per row, spacing at ~20 × 20 cm, in Lingshui (LS; N 18°32′, E 110°01′) and Hangzhou (HZ; N 30°15′, E 120°12′) in 2014.

Rice genomic DNA was extracted from young leaf tissue and all accessions were genotyped using an Illumina custom-designed array containing 5,291 SNP markers following the Infinium HD Assay Ultra Protocol [https://support.illumina.com/downloads/infinium_hd_ultra_assay_protocol_guide_(11328087_b).html] (Illumina, Inc., San Diego, CA, USA) (Lu et al., 2015). Genotypes were called using the GenomeStudio software (Illumina, Inc.). The quality of each SNP was checked manually following a previous study (Yan et al., 2010). SNPs with tri- and tetra-allelic, low quality, high heterozygosis or a minor allele frequency (MAF) <5% were removed from the dataset. Finally, a total of 3,948 SNPs were chosen for further analysis (Table S2).

### Seed germination evaluation and data analysis

The materials were cultivated under the same environmental conditions as much as possible. The heading date of each plant was recorded using the emergence of the first panicle from the flag leaf sheath as a standard. To reduce the marginal effect, four plants in the middle of each line were chosen as testers. The degree of seed dormancy was evaluated using the germination at seed maturity. To minimize the influence of conditions, ~100 filled seeds per line were harvested on the 30–40th day (in the case of the accessions) after heading, and then ~100 seeds were stored at 4°C to maintain seed freshness for germination evaluation on the next day and another ~100 seeds per line were air-dried in greenhouse (~35°C) for 7 days and then treated at 45°C for 3 days to break dormancy. To test the germination, the seeds were wrapped in doubled sheets of 20 × 20 cm wetted absorbent filter paper, placed vertically, and germinated at 30°C and 100% relative humidity in the dark for 10 days. Germinated seeds were defined as those in which the length of shoot exceeded half the length of the seed. Germination (%) = number of germinated seeds × 100/number of tested seeds. This experiment was repeated three times. The mean values of the three replications were used as the final phenotypic data. All seeds were intact grains.

Means, standard errors (SEs), broad-sense heritability ($H_{B}^{2}$), the percentage of phenotypic variation explained by population structure ($R_{Q}^{2}$) and interactions of genotype × environment (G × E) were calculated as in our previous report (Lu et al., 2015). The skewness, kurtosis and coefficient of variation (CV) were calculated using the functions Skew (), Kurt () and STDEV ()/AVERAGE () in Excel 2007, respectively.

### Genome-wide association analysis

To minimize the effects of environmental variation, best linear unbiased predictions (BLUPs) were performed using the R package lme4 (Bates et al., 2011) to estimate the phenotypic value for each line in the two environments (LS and HZ). The BLUP model can be described as

$$\begin{array}{l} Y_{ijk}=L_{k}+E_{i}+R{\left(E\right)}_{ij}+{\left(L\;\times \;E\right)}_{ik}+\varepsilon_{ijk}, \end{array}$$

where *Y*_*ijk*_ is the observed phenotype for the *k*th line in the *j*th replicate of the *i*th environment; *L*_*k*_ is the random effect of the *k*th line; *E*_*i*_ is the random effect of the *i*th environment; *R* (*E*)_*ij*_ is the random effect of the *j*th replicate in the *i*th environment; (*L* × *E*)_*ik*_ is the random interaction effect of the *i*th environment and the *k*th line, and ε_*ijk*_ is a random error following N (0, $\sigma_{e}^{2}$).

GWASs were performed in TASSEL version 4.0 (Bradbury et al., 2007). The EMMA (Kang et al., 2008) and P3D (Zhang et al., 2010) algorithms were used to reduce computing time. The compressed mixed linear model (cMLM) with the population structure matrix (Q) and the relative kinship matrix (K) as covariates was used to reduce false-positive associations (Yu et al., 2006; Zhang et al., 2010). The Q matrix was calculated in our previous study (Lu et al., 2015) and the K matrix was generated using TASSEL version 4.0 (Bradbury et al., 2007) based on the 3,948 SNP markers. Four models including GLM, Q, K, and Q + K were used to evaluate type I errors (GLM model: no correction of population structure or familial relatedness; Q model: the population structure factor (Q matrix) was used as a covariate; K model: the familial relatedness effect (K matrix) was used as a covariate; Q + K model: both Q and K matrixes were used as covariates). The GLM and Q models were implemented using a general linear model program. However, the K and Q + K models were implemented in a mixed linear model program (Lu et al., 2015). The significance threshold for trait-marker associations was determined by Bonferroni correction (α = 1; 1/3,948 = 2.5E-04) (Duggal et al., 2008; Yang et al., 2014). The Bonferroni-corrected threshold probability based on individual tests is calculated to correct for multiple comparisons, using 1/N (α = 1), where N is the number of individual trait-SNP combinations tested. Candidate genes were predicted within a 200-kb genomic region (±100 kb of each significant SNP) from the Rice Haplotype Map Project Database (http://202.127.18.221/RiceHap2/) (Wang C. H. et al., 2014; Lu et al., 2015).

### Candidate gene expression profiles

The expression profiles of all candidate genes were analyzed according to the results of Davidson et al. (2012). Previous studies showed that seed dormancy-related genes had higher expression levels in the embryo, endosperm or seed (Sugimoto et al., 2010; Gu et al., 2011; Ye et al., 2015); thus, the expression data of Davidson et al. (2012) for these three tissues were used to screen the candidate genes. The screened candidate genes were then validated using the expression data in the Bio-Analytic Resource Plant Biology (BAR) database (http://bar.utoronto.ca/) and the Rice Genome Annotation Project database (http://rice.plantbiology.msu.edu/). SNPs located in the candidate genes and haplotype data were obtained from the RiceVarMap database (http://ricevarmap.ncpgr.cn/). Homologous gene identification was performed in the Rice Genome Annotation Project database, and then protein sequences were aligned using BLASTP in NCBI (https://www.ncbi.nlm.nih.gov/).

### Linkage disequilibrium decay, population genetics and polymorphism information content analyses

To detect the regions of improvement sweeps, the 266 landraces and 89 improved lines were used to analyze LD decay, population structure and PIC ratios. The LD decay of the two panels was investigated using TASSEL version 4.0 (Bradbury et al., 2007). The LD decay rate was measured as the chromosomal distance at which the average pairwise correlation coefficient (*r*^2^) dropped to half of its maximum value (Huang et al., 2010; Lu et al., 2015). A neighbor-joining (NJ) tree and principal component analysis (PCA) were used to infer the population structure. Based on Nei's genetic distance (Nei, 1972), the NJ tree was constructed using Powermarker 3.25 (Liu and Muse, 2005) and the PCA was performed using NTSYSpc version 2.1 (Rohlf, 2000). The *F*_ST_ among different subgroups and the PIC of each SNP marker were also evaluated using Powermarker 3.25 (Liu and Muse, 2005). The PIC ratio was used to test the improvement sweep regions according to differing genetic diversity of the two populations in the selected regions of the genome. The equation for the ratio can be expressed as follows: PIC ratio = PIC_(landrace)_/PIC_(improvedline)_. SNPs with PIC ratios > 3 were empirically considered improvement footprints.

## Results and discussion

### Diversity panel and phenotypic variation

**Figure 1:**
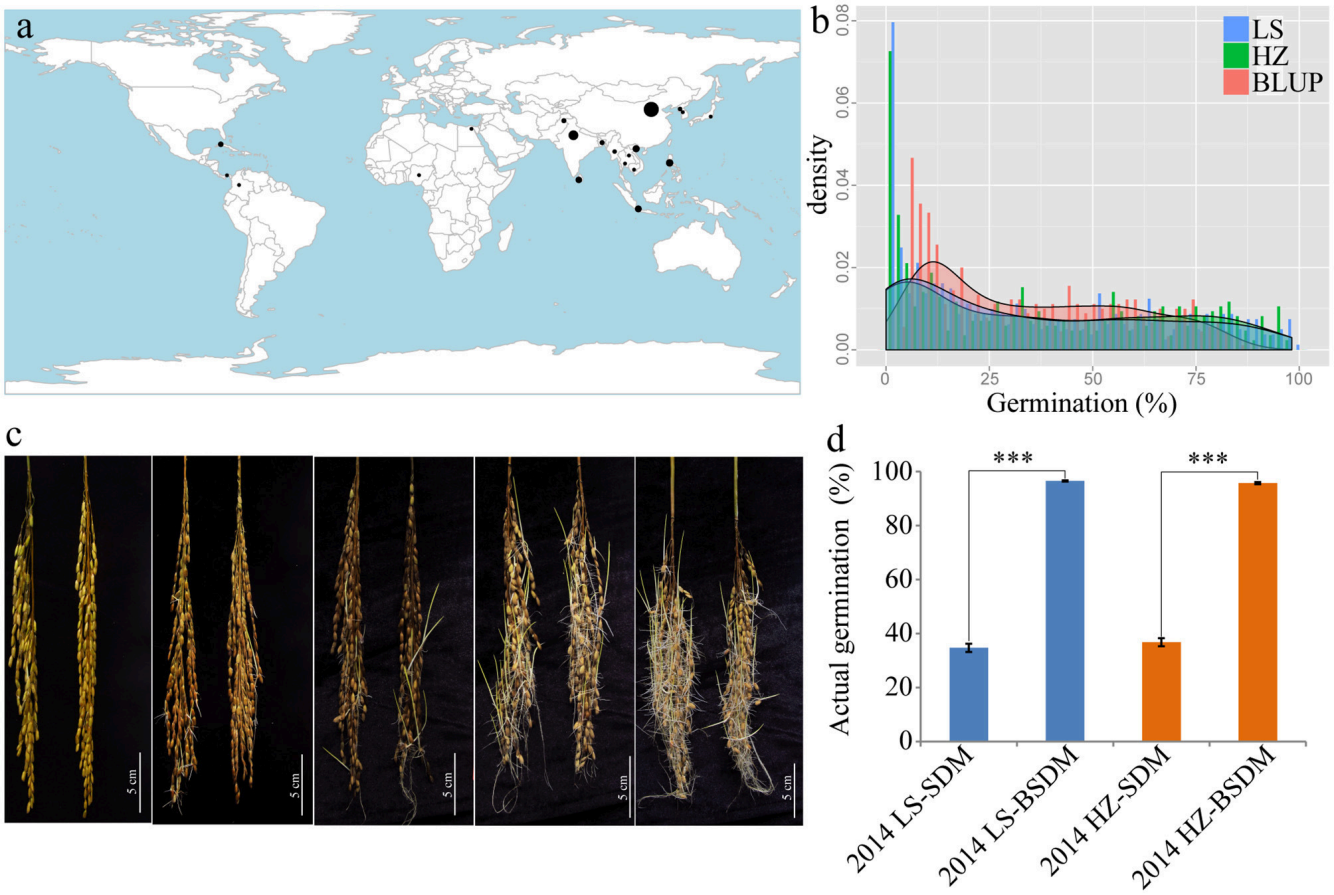
Material distribution and seed dormancy diversity. **(a)** The worldwide distribution of 453 *indica*-only accessions. **(b)** Histogram of pre-harvest sprouting in Lingshui (blue), Hangzhou (green) and the BLUP phenotypes (red). **(c)** Differences in seed germination in the germplasm. Bar = 5 cm. **(d)** Seed actual germination comparison before and after breaking dormancy in two environments. SDM, seed dormancy; BSDM, breaking seed dormancy; LS, Lingshui; HZ, Hangzhou. ***P = 0.001.

A rice diversity panel consisting of 453 *indica* accessions gathered from 20 rice-planting countries (Figure 1a) was used in our GWAS analyses. These accessions have rich seed dormancy variation, from deep to weak dormancy (Figures 1b,c). The germination of dormant seeds and dormancy-broken seeds were used to confirm the existence of dormancy. The germination of dormancy-broken seeds was nearly 100%, which was significantly higher than that of dormant seeds regardless of environment (LS or HZ), indicating that all of the accessions had true dormancy characteristics and were viable (Figure 1d).

Germination of threshed seeds was used to test the degree of seed dormancy in our study. The G × E analysis indicated that environmental effects should not be ignored (Table 1). Thus, to minimize the effect of environment variation, BLUPs of the genetic effect for each line were used for the overall association analysis in the panel. The phenotypic variations of germination of threshed seeds in the two environments and predictions using the BLUP method are shown in Table 1 and Table S3. The mean germination were 34.7, 36.8, and 34.8% in LS, HZ and the BLUPs, respectively. The coefficients of variation were 87.8, 84.3, and 67.3%, respectively. Moreover, the ranges of germination were 0.0–98.2%, 0.0–97.4%, and 5.8–86.0%, respectively. These results suggested that the accessions had abundant phenotypic variation and were suitable for GWAS analysis (Pearson and Manolio, 2008). The positive skewness value ranged from 0.4 to 0.5, suggesting that the germination had a certain skewed distribution (Table 1, Figure 1b). The phenotypic variation explained by the population structure ($R_{Q}^{2}$) ranged from 45.7 to 61.2%; additionally, the broad-sense heritability ($H_{\text{B}}^{2}$) was 85.2%.

**Table 1:** Phenotypic variation of pre-harvest seed germination in 453 *indica* accessions in two environments.

**Trait**	**Germination (%)**		
**Env**.	**2014-LS**	**2014-HZ**	**BLUP method**
Mean	34.7	36.8	34.8
SE	1.5	1.5	1.1
Min	0.0	0.0	5.8
Max	98.2	97.4	86.0
CV%	87.8	84.3	67.3
Skewness	0.5	0.4	0.4
Kurtosis	−1.1	−1.3	−1.1
${\text{H}}_{Q}^{2}$ %	45.7	61.2	60.0
${\text{H}}_{\text{B}}^{2}$ %	85.2		
G^*^E	^***^		

*** *P = 0.001*.

Rice has been found in archaeological sites dating to 8000 B.C. (Higham and Lu, 1998) and has been domesticated artificially and naturally from wild rice over a long period. Great changes have occurred in numerous traits, such as seed dormancy. Thus, one possible explanation for the skewed phenotype distribution was that the degree of seed dormancy in some cultivated rice accessions was significantly weakened to meet production needs during rice domestication (Sweeney and McCouch, 2007). The high $R_{Q}^{2}$ value suggested that the phenotypic variation was strongly affected by the population structure of the panel (Lu et al., 2015), and in further GWAS analyses, the population structure factor (Q matrix) should be taken into consideration to adjust the GWAS results. In addition, the relatively high $H_{\text{B}}^{2}$ indicated that genetic improvement of the trait was effective and could play a significant role in the breeding process in the future.

In recent years, GWASs in plants have become a new QTL detection strategy to unlock the genetic secrets of heritable traits through high-throughput genotyping technologies based on a large natural germplasm collection containing rich genetic and phenotypic variation (Huang et al., 2010). Compared with linkage mapping, GWASs use a germplasm mapping population and LD to improve the genetic mapping efficiency and mapping precision (Remington et al., 2001; Salvi and Tuberosa, 2005). Here, a large *indica* rice diversity panel containing a wealth of genetic diversity in seed dormancy (Figures 1b,c, Table 1) was used to perform a GWAS. Previous studies have also demonstrated that this *indica*-only population is well suited for association mapping (Lu et al., 2015, 2016).

### Genome-wide analysis of seed dormancy

**Figure 2:**
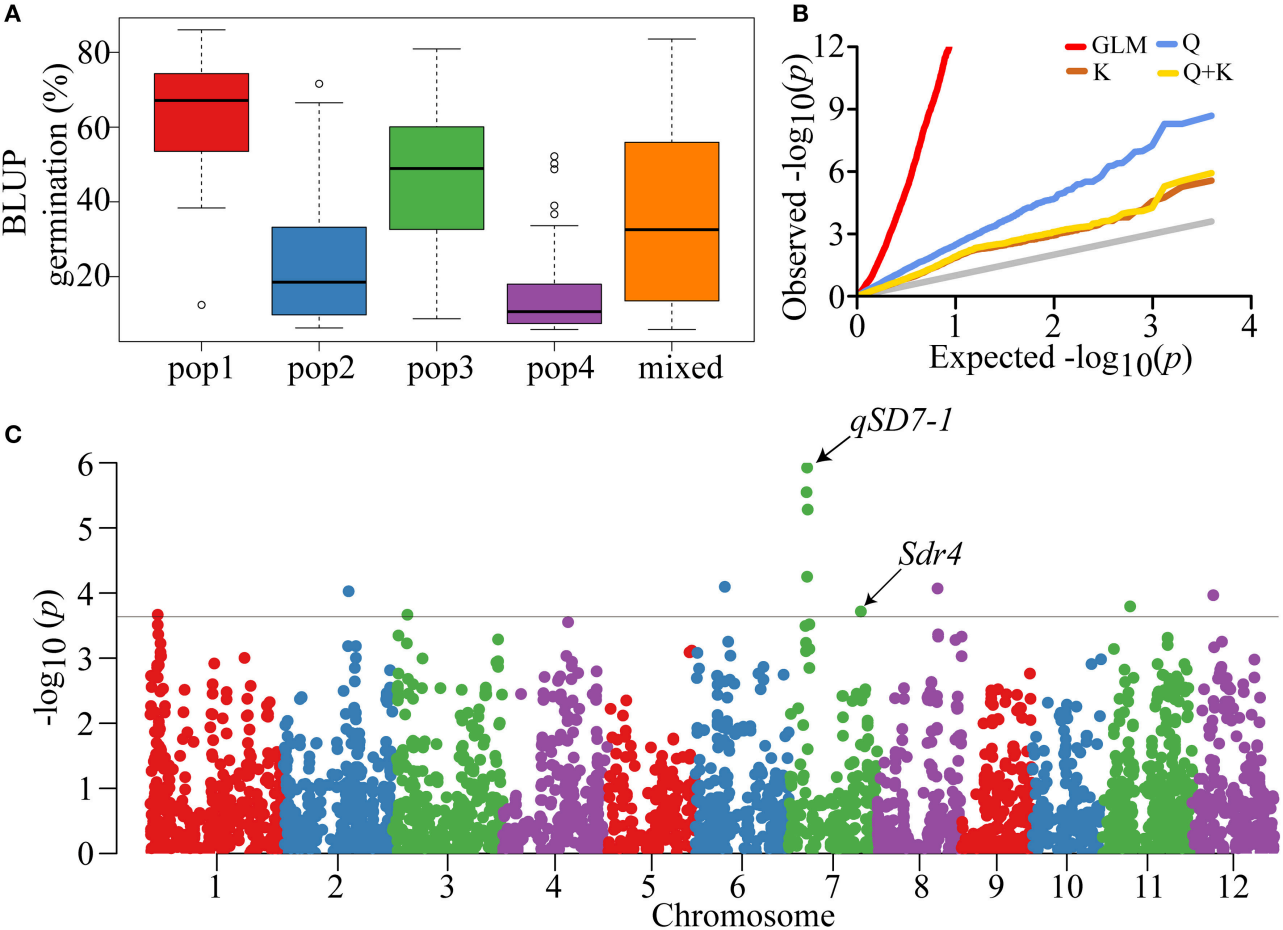
Phenotypic distribution and GWAS of seed dormancy. **(A)** Boxplot showing the differences in seed germination among five subpopulations of the *indica*-only panel. To reduce environment affect and simplify analysis, BLUP of seed germination of each subpopulation was used in the boxplot. Whiskers represent 1.5 times the quantile of the data. Individuals falling outside the range of the whiskers are shown as open dots. **(B)** Quantile-Quantile plots of GWAS results using different association models for all samples. Gray line represents the distribution of *P*-values assuming associations. **(C)** Manhattan plot showing *P*-values along the whole genome. The black straight line shows the threshold of *P* = 2.5E-04.

Our previous report showed that the *indica*-only panel could be classified into four populations (POPs) and a mixed subgroup (Mixed) (Figure S1), and relative kinship analysis indicated that there was no or weak relatedness in the panel (Lu et al., 2015, 2016). Each accession was assigned to a corresponding group using Q component ≥ 0.6 as a threshold. The average germination was significantly different among the five populations (Figure S1). In particular, POP1 had the highest value (>60%) and POP4 had the lowest (~15%) (Figure 2A). This result was also supported by the high $R_{Q}^{2}$ (Table 1). Taken together, these results highlighted the need to account for population structure and relative kinship when performing the subsequent GWAS analyses.

To evaluate the effects of the two elements and control false positive associations, four models (GLM, Q, K, and Q + K) were compared using a quantile-quantile (Q − Q) plot (Figure 2B). Compared with the GLM and Q models, the K and Q + K models showed great control of type I errors. Moreover, the Q + K model included both population structure and relatedness. Therefore, all further analyses were performed using the Q + K model with the cMLM.

The GWAS was conducted using the BLUPs of individual germination over the two environments. Through the GWAS, a total of 12 SNPs significantly associated with the seed dormancy trait were detected across eight chromosomes (Figure 2C). Four significant SNPs located close to each other were detected on the short arm of the chromosome 7 within a 260-kb region, among which the lead SNP (seq-rs3227, *P* = 1.19E-06) was used as the representative. As a result, nine peak SNPs were retained (Table 2). The contribution of a single SNP to the phenotypic variation ranged from 3.5 to 5.8%, and together the SNPs explained 34.9% of the variation (Table 2). In addition, all SNPs except seq-rs5598 on chromosome 12 were adjacent to or overlapped with previously reported QTLs (Table 2). In particular, the strongest trait-associated SNP, seq-rs3227 (*P* = 1.19E-06), on chromosome 7 was located ~164.2 kb downstream of the cloned pleiotropic gene *qSD7-1* for seed dormancy, and another peak SNP, seq-rs3527 (*P* = 1.93E-04), resided ~3.7 kb downstream of *Sdr4*, which is involved in seed dormancy (Figure 2C; Figure S2) (Sugimoto et al., 2010; Gu et al., 2011).

**Table 2:** Summary of lead SNPs significantly associated with the seed dormancy trait.

**Sig. SNPs**	**Allele**	**Chr**.	**Position (IRGSP v4)**	***P* value**	***R*^2^ (%)**	**Total *R*^2^ (%)**	**Known QTLs**
seq-rs81	T/C	1	2,580,387	2.40E-04	3.5	34.9	RM6902 (Marzougui et al., 2012)
seq-rs1000	T/C	2	21,983,557	9.35E-05	3.9		RM525-RM318-RM240 (Xie et al., 2011)
seq-rs1445	G/A	3	5,178,318	2.36E-04	3.5		RM231 (Wan et al., 2006)
seq-rs2896	C/A	6	10,198,431	7.96E-05	4.0		RM5963 (Marzougui et al., 2012); RM549 (Gu et al., 2004)
seq-rs3227	G/A	7	6,265,668	1.19E-06	5.8		*qSD7-1* (Gu et al., 2011); RM180 (Gu et al., 2004)
seq-rs3527	C/A	7	24,461,489	1.93E-04	3.6		FHS7.0 (Magwa et al., 2016); *Sdr4* (Sugimoto et al., 2010); RM234 (Wan et al., 2006); RM346 (Gu et al., 2004)
seq-rs3939	A/G	8	20,453,214	8.44E-05	4.0		FHS8.1 (Magwa et al., 2016); RM531 (Gu et al., 2004)
seq-rs4917	A/G	11	9,746,917	1.60E-04	4.1		*qSD-11* (Guo et al., 2004)
seq-rs5598	A/C	12	7,132,922	1.07E-04	3.9		

In recent years, association studies have become a leading method to detect genes (or QTLs) underlying human diseases (Edwards et al., 2005) and agriculturally complex traits (Huang et al., 2010). However, the inflation of type I errors (false positive associations) caused by gene effects, allele frequencies, sample size and marker density is inevitable (Pe'er et al., 2006; Moonesinghe et al., 2007). Population structure and relative kinship are two common factors that determine the false positive rate (Zhang Z. et al., 2009). In our study, the high $R_{Q}^{2}$ (Table 1), which was supported by the large germination variation among the five different populations (Figure 2A), indicated that population stratification could cause some false positive associations when performing GWAS analyses. Thus, the cMLM (Q + K) (Zhang et al., 2016b) method was used to effectively eliminates false positive results by combining the two covariates simultaneously (Figure 2B).

In previous studies, many seed dormancy QTLs distributed on the 12 rice chromosomes have been identified using traditional linkage mapping (Gu et al., 2004, 2011; Guo et al., 2004; Wan et al., 2006; Sugimoto et al., 2010; Xie et al., 2011; Marzougui et al., 2012). In this study, the phenotypic variation explained by each individual significant SNP was <6%. This result demonstrated that rice seed dormancy is a typical quantitative trait with a minor genetic effect. Moreover, eight of the nine associated SNPs were detected previously in linkage analyses (Table 2) and two cloned seed dormancy genes, *qSD-7-1* and *Sdr4* on chromosome 7, were adjacent to the peak trait-associated SNPs seq-rs3227 and seq-rs3527, respectively (Table 2). Thus, the GWAS lead SNPs were largely confirmed by previous reports, showing that our results were reliable and implying that genome-wide association mapping is an effective strategy to uncover novel QTLs for complex agronomic traits based on a high density genetic map, although only one SNP, seq-rs5598 (*P* = 1.07E-04), was not detected in previous reports.

Seed dormancy is easily affected by multiple environmental factors, and seed maturing time is one of the most important influencing factors. However, it is difficult to investigate because there is no effective standard to define or evaluate when the seeds are mature. Generally speaking, seed that turns yellow and hardened means maturity. But this is a continuous process of changes and it is hard to record a specific time. In this study, we harvested filled seed on the 30–40th day after heading in the case of each accession by empiricism to reduce the effects of seed maturing time. In conclusion, the relationship between seed dormancy and seed maturing time should be explored in depth. In addition, how to define and determine seed maturing time that may be related to grain-filling rate will also be a valuable topic to deep study in future.

### Trait-associated SNPs effects and molecular breeding application

**Figure 3:**
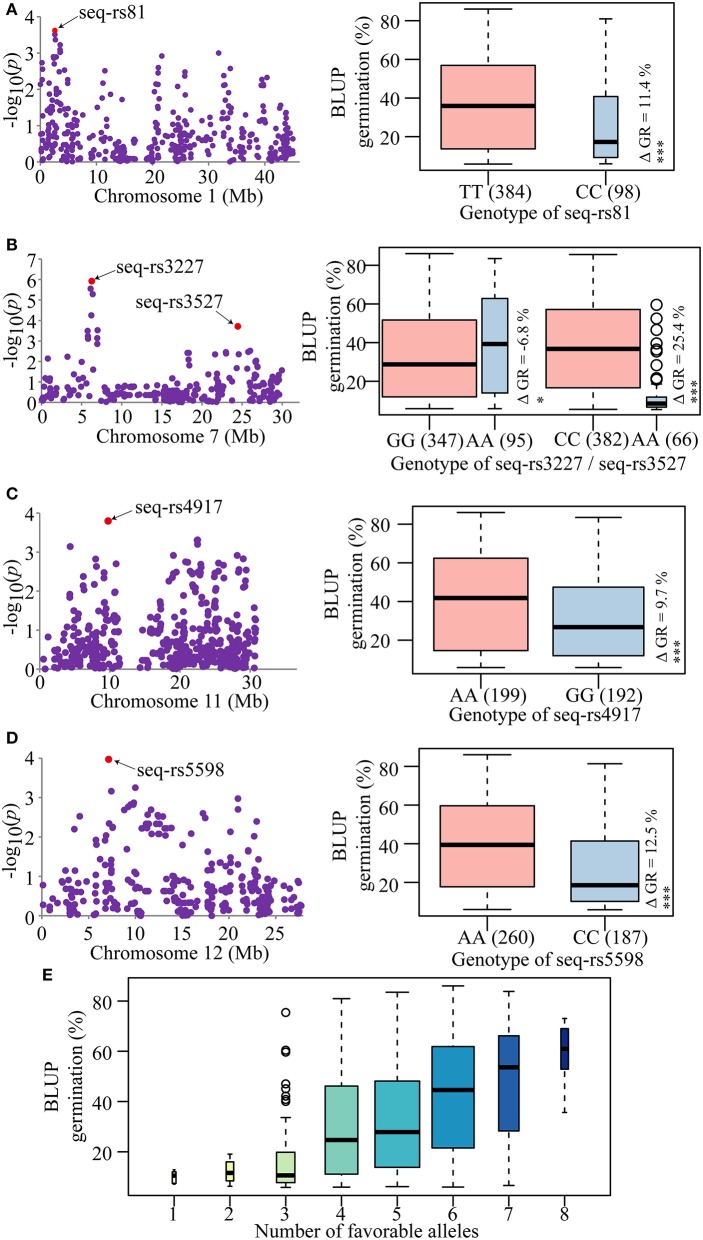
Trait-associated SNPs and pyramiding of favorable alleles. **(A–D)** Candidate SNPs associated with seed dormancy and phenotypic differences between the two alleles of each SNP. Red dots represent the lead SNPs of candidate regions. The boxplots on the right show the distribution of average germination estimated by BLUP for each SNP allele. The number of individuals for each allele is given in parenthesis and is represented by the width of the box. Whiskers represent 1.5 times the quantile of the data. Individuals falling outside the range of the whiskers are shown as open dots. Differences in means are shown by ΔGR. ^*^P = 0.05 and ^***^P = 0.001, respectively. **(E)** Pyramid effects for different numbers of favorable alleles of the candidate SNPs. The X-axis represents the number of elite alleles carried by the accessions and the Y-axis represents the trait mean value estimated by BLUP. The width of each boxplot represents the number of accessions.

The phenotypic differences between the two alleles of each of the strongest trait-associated SNPs are summarized in Table S4. Using the absolute value as the standard, the deviation values ranged from ~0.67 to ~25.4% for each SNP. Moreover, the deviation values of five SNPs reached significant (*P* ≤ 0.05) or highly significant (*P* ≤ 0.01 or 0.001) levels between the two alleles of each SNP marker (Figure 3). Among them, the absolute values of four SNPs were close to or more than 10%, especially for the seq-rs3527 (*P* = 1.93E-04), which was ~25.4% (Figure 3B; Table S4). The results suggested that all these trait-associated SNPs could be used efficiently for molecular breeding in the future.

To confirm this, the elite alleles with positive effects were used to test the effectiveness of pyramid breeding. Without considering the effects of interactions among these lead SNPs and environmental influences, the more elite alleles that were pyramided in a variety, the higher the germination increase (Figure 3E). This result indicated that pyramiding of favorable alleles could attenuate seed dormancy. In practice, according to their breeding goals, breeders could introduce different numbers of favorable alleles into different varieties to modify the seed dormancy trait. In the GWAS panel, most of the accessions carried four to six favorable alleles and had moderate germination ranging from 30.8 to 42.4%. Only a few of accessions had an extreme germination; <18.1% or larger than 48.3% (Figure 3E). This result suggested that a suitable number of favorable alleles have been maintained in these accessions through artificial phenotypic selection in the process of rice breeding to meet cultivation needs. Accessions with extreme phenotypes (very high or low PHS) do not suit human needs and are gradually eliminated during the breeding process, but these accessions may be good materials for genetic research. Since LS and HZ are of different latitudes, the day-length and temperature are quite different. Which environment would be more suitable for the favorable allele mining for the *indica* breeding practice? Alleles favorable under both locations were stably expressed would be widely adopted by more breeders throughout the country. However, those mined specifically at one location would also offer useful information for the breeding work under similar conditions. For example, the changes of day-length and temperature are more obvious in HZ other than that in LS throughout the year. Consequently, the phenotypic effects of some favorable alleles would be more apparent and easier to observe, especially for those that sensitive to the day-length and temperature.

How can these useful lead SNPs be applied to molecular breeding? Although the flanking sequence of each SNP (Table S2) can be used to detect the SNPs for breeding with next-generation sequencing technology, to some extent, this method is inconvenient. Thus, a dCAPS marker for each SNP was designed using dCAPS Finder 2.0 (Neff et al., 2002) (Table S5). These dCAPS markers can clearly distinguish the genotypes of the corresponding SNPs in polypropylene gel electrophoresis (Figure S3). Generally speaking, these markers will be beneficial for molecular marker-assisted selection breeding in the future.

### Candidate gene prediction and expression profiling

The flanking regions within a 200-kb window (±100 kb) of the lead SNPs were searched to identify candidate genes in the Rice Haplotype Map Project Database (Wang C. et al., 2014; Lu et al., 2016). For the two SNPs on chromosome 7, seq-rs3227 (*P* = 1.19E-06) and seq-rs3527 (*P* = 1.93E-04), two known seed dormancy-related genes, *qSD7-1* (Gu et al., 2011) and *Sdr4* (Sugimoto et al., 2010), respectively, were located in the corresponding region (Table 2, Figure S2). For the other seven SNPs, a total of 212 candidate genes were identified, which are summarized in Table S6.

**Figure 4:**
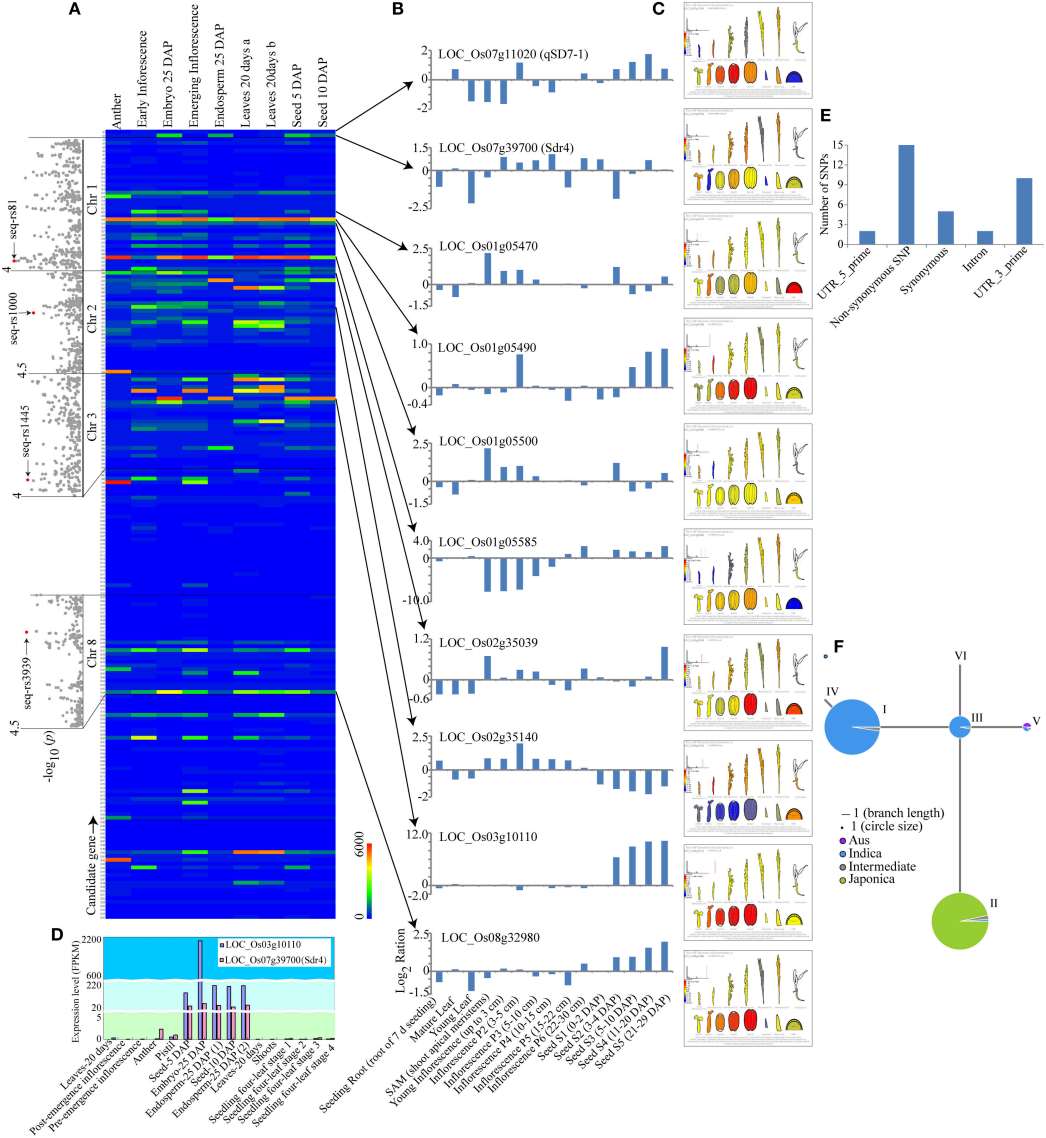
GWAS analysis and candidate gene identification**. (A)** Genome-wide association signals and expression levels of the predicted genes of the lead SNPs. The positions of the peak SNPs are indicated by red dots. The expression level of each predicted gene is shown in the color index at the bottom right of the panel. **(B)** Expression pattern comparison between eight candidate genes and two known genes, *qSD7-1* and *Sdr4*. **(C)** The expression pattern of each gene according to the Bio-Analytic Resource for Plant Biology expression database. Red and blue represent high and low relative expression levels in different tissues, respectively. **(D)** Gene expression pattern comparison between the candidate gene LOC_Os03g10110 and the known gene *Sdr4* in different stages of seed development. **(E)** The presence of SNPs in the candidate gene LOC_Os03g10110 according to the public RiceVarMap database. **(F)** Haplotype network analysis using the public RiceVarMap database. Blue and green represent *indica* and *japonica* accessions, respectively.

To identify the most promising candidate genes, the expression levels in embryo, endosperm and seed tissues, which were downloaded from the results of Davidson et al. (2012), were used as a screening reference. Interestingly, *qSD7-1* had a high expression level in the seed at 5 days after pollination (DAP). By comparison, the *Sdr4* had high expression levels in the embryo at 25 DAP, endosperm at 25 DAP, and seeds at 5 and 10 DAP (Figures 4A,B, Table S6). These results were consistent with previous reports (Sugimoto et al., 2010; Gu et al., 2011; Ye et al., 2015). After searching the expression data for the 212 candidate genes, the expression patterns of eight genes were found to be similar to those of *qSD7-1* or *Sdr4* (Figures 4A,B, Table S6). Then, expression data from the BAR database and the rice genome annotation project database were used to verify the eight genes (Figure 4C, Table S6). The candidate gene LOC_Os03g10110, which encoded a cupin domain-containing protein, had high expression levels in seed tissues at different stages (Figures 4B,C, Table S6). Moreover, the expression pattern of this gene was highly consistent with that of *Sdr4*, which has very high expression levels in seeds at 5 and 10 DAP, embryos at 25 DAP, and endosperm at 25 DAP (Figure 4D, Table S6). Homology analysis indicated that the gene was homolog to grmzm2g078441, a gene in the cupin family of unknown function that is highly expressed in embryo tissues in maize (Teoh et al., 2013). A previous report suggested that cupins comprise a superfamily of functionally diverse proteins that include germins and plant storage proteins (Dunwell, 1998). Storage proteins may be important in plants during seed germination and seedling growth (Shewry et al., 1995). The RiceVarMap database (http://ricevarmap.ncpgr.cn/) showed that a total of 34 SNPs were located within this gene (Table S7) and that 15 SNPs were non-synonymous mutations (Figure 4E). Haplotype network analysis indicated that most *indica* accessions could be classified into haplotype groups I and III, while the majority of *japonica* accessions were assigned to haplotype group II (Figure 4F, Table S8). This result suggested that the haplotype of the candidate gene displayed some *indica*-*japonica* specificity.

### Population division, nucleotide diversity and linkage disequilibrium between improved lines and landraces

Rice improvement is the outcome of continuous artificial selection to enhance the adaptation of the plant to fit human needs. Consequently, various rice traits, such as plant type and seed shape, have been changed dramatically (Figures S4A,B). The successive selection effect also alters the genetic diversity at the genomic level, resulting in a distorted pattern of genetic variation and LD decay.

**Figure 5:**
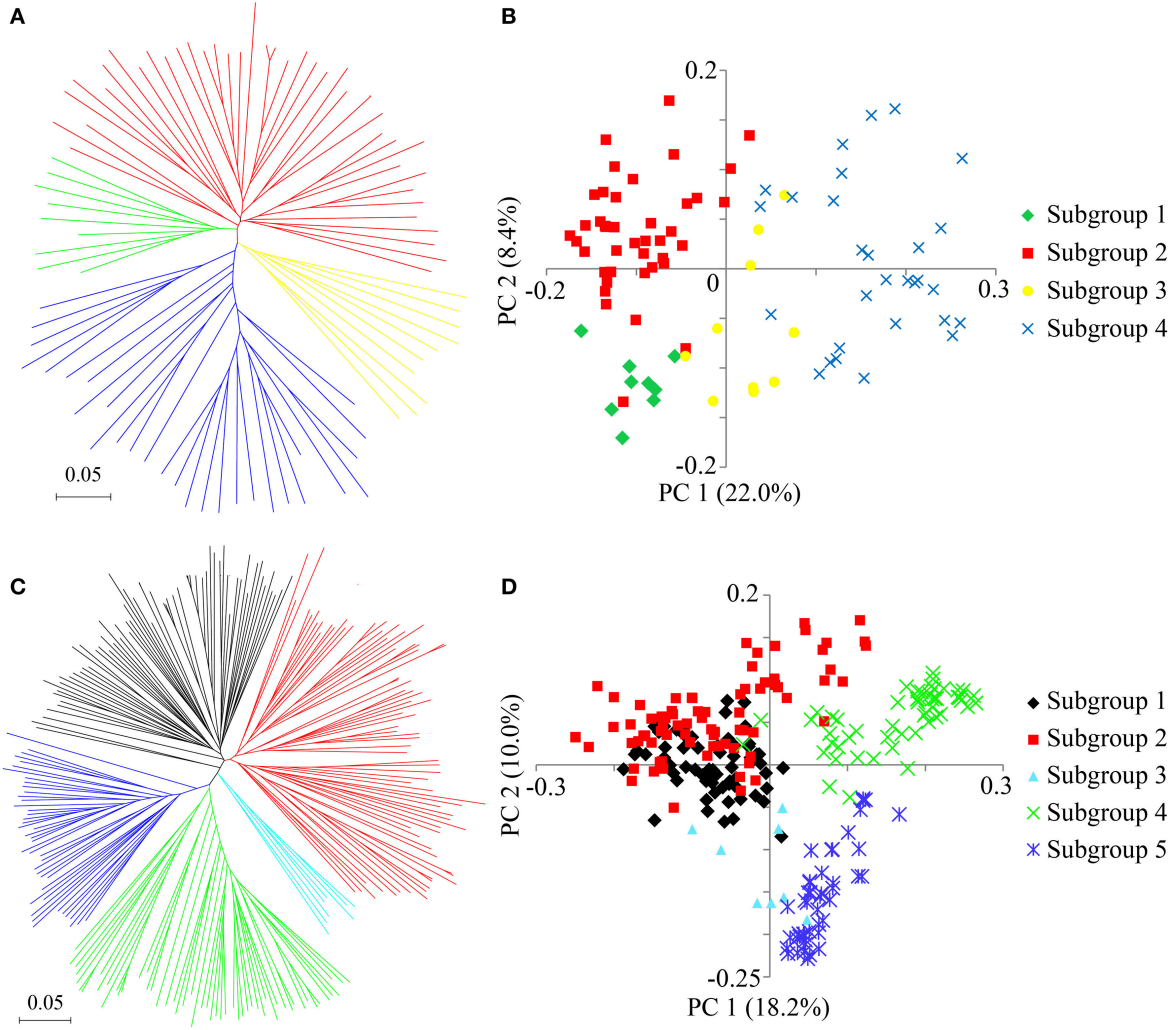
Population structure of the improved lines and landraces**. (A)** Neighbor-joining tree of 89 improved lines. **(B)** PCA plot of the first two components of the 89 improved lines. **(C)** Neighbor-joining tree of 266 landraces. **(D)** PCA plot of the first two components of the 266 landraces. The subgroups identified from the neighbor-joining tree are color-coded in each corresponding PCA plot.

To identify selective sweeps across the whole rice genome, the 89 improved lines and 266 landraces, most of which were widely distributed in southern China, were used to detect selective sweep signals (Figures S4C,D). To better understand the population stratification and geographic structure diversity, a NJ tree was constructed and PCA was performed to illustrate the relatedness among the accessions. The results indicated that the improved lines comprised four subgroups, among which PC1 and PC2 accounted for 22.0 and 8.4% of the genetic variation, respectively (Figures 5A,B). However, the landraces were classified into five subgroups, among which PC1 and PC2 explained 18.2 and 10.0% of the genetic variation, respectively (Figures 5C,D). The *F*_ST_ ranged from 0.07 to 0.47 among all nine subgroups, indicating strong population differentiation among some of the subgroups (Figure S5). The *F*_ST_ averaged 0.17 among the subgroups of the improved lines, suggesting a moderate level of differentiation, and was estimated at 0.26 on average among the landrace subgroups, implying greater population differentiation than in the improved lines (Figure S5). These averages were close to previously published values in rice (Wang C. H. et al., 2014; Lu et al., 2015), but much less than that between *indica* and *japonica* (*F*_ST_ = 0.55) (Huang et al., 2010), and a little bit larger than in soybean (Wen et al., 2014), maize (Yang et al., 2011), and sesame (Wei et al., 2015).

**Figure 6:**
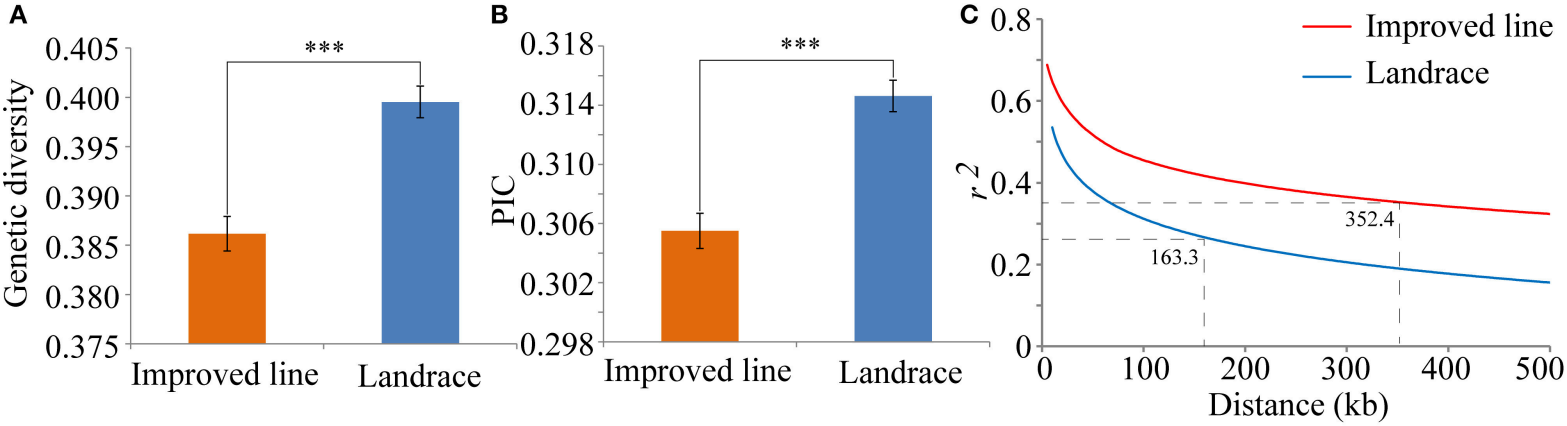
Characteristics of SNP diversity and genome-wide average LD decay in two panels. **(A)** Genetic diversity of SNPs in the improved lines and landraces. **(B)** Polymorphism information content in the improved lines and landraces. **(C)** Estimation of genome-wide average LD decay distances from the improved line (red) and landrace panels (blue). The dashed line represents the LD decay rate, which was measured as the chromosomal distance at which the average pairwise correlation coefficient (*r*^2^) dropped to half of its maximum value. ^***^P = 0.001.

To further evaluate the degree of human selection, the gene diversity, PIC and LD decay were quantified for the two populations. Gene diversity and PIC values for the landraces were both significantly higher than in the improved lines (Figures 6A,B). These estimations were close to previous estimates from 926 SNPs in maize (Yang et al., 2011), but much smaller than those calculated from simple sequence repeat (SSR) markers in rice (Wang C. H. et al., 2014) and spring barley landraces (Pasam et al., 2014). This result demonstrated that the landraces had retained more genetic diversity than the improved lines. Because increased LD is another hallmark of artificial selection in rice, the LD decay rates between the two populations were compared. The extent of LD decay increased from 163.3 kb for the landraces to 352.4 kb for the improved lines (Figure 6C). The larger LD decay distance in the improved lines may have been caused by a loss of genetic diversity and a low frequency of genetic recombination because of human selection forces during improvement (Lam et al., 2010). The extent of LD for the landraces was similar to that in a previous evaluation in rice (Huang et al., 2010; Lu et al., 2015), but much greater than that in maize (Remington et al., 2001).

### Genome wide selective sweep signal scan

**Figure 7:**
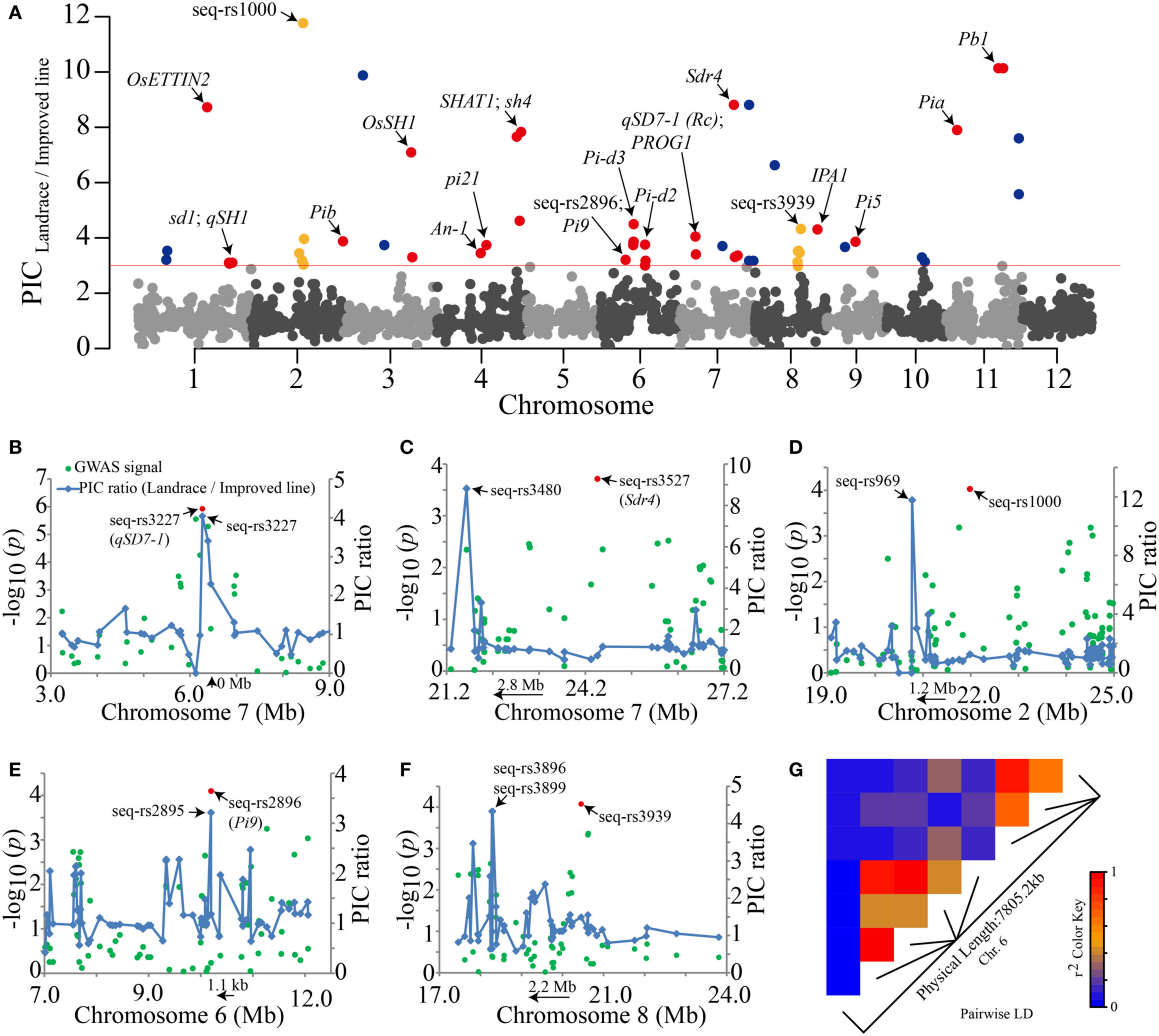
Visualization of genome-wide selection signals and the GWAS results. **(A)** Whole-genome screening of selection signals from rice improvement. The PIC ratio values are plotted against their positions on each of the 12 chromosomes. The red horizontal line indicates the threshold (PIC ratio = 3) for the selection signals. Previously reported genes, our GWAS lead SNPs and new putative selective sweeps (Table S9) are marked with red, yellow and dark blue dots, respectively. **(B–F)** Five GWAS results, including the two seed dormancy genes *qSD7-1* and *Sdr4*, that are very close to strong selection signals. The peaks of blue lines and red dots represent selective sweep regions and GWAS signals, respectively. The orientations of the two known genes and selective sweep regions are indicated by arrows. **(G)** The extent of LD around the SNPs in multiple resistance genes on chromosome 6. The *r*^2^ values are indicated by the color key.

Artificial selection has probably changed the nucleotide diversity within the genomes of cultivars. To detect the genomic regions most affected by artificial selection during rice improvement, PIC ratios between the landraces and improved lines were screened throughout the whole genome (Figure 7A). PIC ratios >3.0 (the top ~1% of all values) were retained as an empirical threshold. After screening, the PIC ratios of 57 SNPs exceeded the cutoff. Peak signals within a ~±1.5-Mb window were grouped into a single DNA region because the selection effect leads to greater LD decay and extended haplotype structure (Qanbari et al., 2014). Finally, 27 selection DNA regions were identified (Table S9). Among these regions, 18 known domestication-related genes resided in 15 corresponding selection regions, such as the “green revolution” gene *sd1* (Sasaki et al., 2002), the seed shattering-related gene *sh4* (Li et al., 2006), *qSD7-1* and *Sdr4* for seed dormancy (Sugimoto et al., 2010; Gu et al., 2011) and *IPA1* for ideal plant architecture (Jiao et al., 2010) (Figure 7A, Table S9).

Interestingly, in our GWAS, the *qSD7-1* gene for seed dormancy was located near the most significant SNP seq-rs3227 (*P* = 1.19E-06) (Table 2). This SNP also showed strong selection signals with a highest PIC ratio value of ~4.0 within a 6-Mb genome region (Figure 7B). Notably, another selection signal, seq-rs3480 (PIC ratio = 8.8), appeared near the seed dormancy-related gene *Sdr4*, which resided upstream of the GWAS signal of seq-rs3527 (*P* = 1.93E-04) (Table 2) within a ~2.8 Mb physical distance (Figure 7C). More importantly, there were three strong selection signals in the vicinity of the corresponding three peak SNP previously identified by the GWAS method (Table 2, Figures 7D–F). In particular, one of the GWAS peak SNPs (seq-rs2896, *P* = 7.96E-05) was located just 1.1 kb downstream of the strongest selection SNPs (seq-rs2895, PIC ratio = 3.2) (Figure 7E). In addition, on chromosome 6, there were eight selection signals adjacent to rice blast resistance genes (Figure 7A, Table S9). The pairwise LD values showed that most of these were not located in one haplotype block (*r*^2^ < 0.8) (Figure 7G), implying that rice resistance-related traits may also have experienced strong artificial selection during rice improvement (Figure 7E).

Taken together, our results suggested most of the selective sweep regions (~66.7%) were adjacent to both known domestication-related genes and SNPs we identified previously by GWAS (Table 2, Figure 7, Table S9). These results indicated that genome-wide screening to detect selective sweeps using PIC ratios between two populations with different degrees of human selection can be used to identify DNA regions potentially related to artificial selection in rice. Additionally, the accuracy of our previous GWAS results (Table 2) was further validated by these selective sweep regions. However, no selection signals were detected on chromosomes 5 and 12. There are two possible reasons for this. On the one hand, a lack of sufficient polymorphic SNP markers in these specific genome regions among the two panels may have led to poor detection capability. On the other hand, weak selective pressure on these two chromosomes may have limited our power to detect selection signals.

## Conclusions

In this study, a total of nine known and new SNPs associated with rice seed dormancy were identified via GWAS. dCAPS markers were designed to accelerate the molecular breeding of rice dormancy. Moreover, 212 candidate genes were identified. The expression profiles and haplotype network data from public databases revealed eight genes, especially LOC_Os03g10110, which has a maize homolog involved in embryo development, as candidate regulators for further investigations to verify their biological functions. A genome-wide screen to detect artificial selection signals identified 27 selection DNA regions. Among them, 15 were adjacent to known domestication-related genes and three strong selection signals were located near GWAS lead SNPs. These results not only further verify the accuracy of our GWAS findings but also suggest that genome-wide screening for selective sweeps can be used to identify new improvement-related DNA regions, although the phenotypes are unknown. This study enhances our knowledge of the genetic variation in rice seed dormancy, and the new GWAS SNPs will provide real benefits for genomic selection in breeding programs. More importantly, the genomic consequences of improvement footprints will enable the detection of domestication-related traits.

## Availability of data and material

The SNP dataset used during this study is available in the in the Dryad digital repository (https://doi.org/10.5061/dryad.cp25h). Any other datasets used and/or analyzed during the current study available from the corresponding author on reasonable request.

## Author contributions

Conceived and designed the experiments: QL, XN, and XW. Performed the experiments: QL, XN, MZ, CW, QX, and YF. Analyzed the data: QL, YY, SW, XY, HY, and YW. Wrote the paper: QL. Revised the manuscript: XC, XL, and XW. All authors read and approved the final manuscript.

### Conflict of interest statement

The authors declare that the research was conducted in the absence of any commercial or financial relationships that could be construed as a potential conflict of interest.

## Abbreviations

ABA: Abscisic acid
BLUP: Best linear unbiased prediction
cMLM: Compressed mixed linear model
CV: Coefficient of variation
DAP: Day after pollination
dCAPS: Derived cleaved amplified polymorphic sequences
EHH: Extended haplotype homozygosity
*F*_ST_: Population differentiation
GA: Gibberellic acid
G × E: Interactions of genotype and environment
GWAS: Genome-wide association study
$H_{\text{B}}^{2}$: Broad-sense heritability
HZ: Hangzhou
LD: Linkage disequilibrium
LS: Lingshui
MAF: Minor allele frequency
NJ: Neighbor-joining
PCA: Principal component analysis
PHS: Pre-harvest sprouting
PIC: Polymorphism information content
QTL: Quantitative trait locus
SE: Standard error
SNP: Single nucleotide polymorphism
SSR: Simple sequence repeat
XP-CLR: Cross-population composite likelihood ratio.
