# Supplementary material for: Genome-Wide Association Study of Seed Dormancy and the Genomic Consequences of Improvement Footprints in Rice (Oryza sativa L.)
Source: Front Plant Sci. 2018 Jan 5;8:2213. doi: 10.3389/fpls.2017.02213 (PMC5760558; doi:10.3389/fpls.2017.02213)
Supplement: Supplementary file 10 [file Image1.PDF]

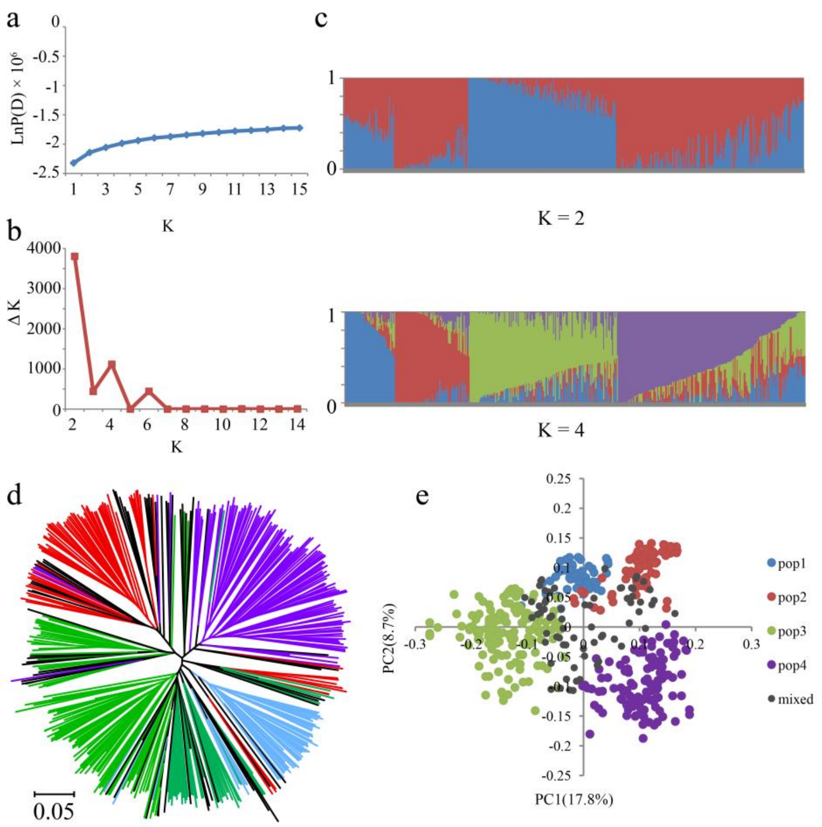

Figure S1 Population structure analyses of the *indica*-only panel. The results showed that the panel can be classified into four populations (pop1 to pop4) and a mixed subgroup (mixed). (a) Mean LnP(D) values; (b) ΔK values; (c) Subpopulations (K = 2 and K = 4) inferred using STRUCTURE; (d) NJ tree based on Nei's genetic distances. Blue, red, green, purple and black represent pop1, pop2, pop3, pop4 and mixed, respectively; (e) Principal component analysis. These results were from our previous reports (Lu et al., 2015).
