# Supplementary material for: Genome-Wide Association Study of Seed Dormancy and the Genomic Consequences of Improvement Footprints in Rice (Oryza sativa L.)
Source: Front Plant Sci. 2018 Jan 5;8:2213. doi: 10.3389/fpls.2017.02213 (PMC5760558; doi:10.3389/fpls.2017.02213)
Supplement: Supplementary file 11 [file Image2.PDF]

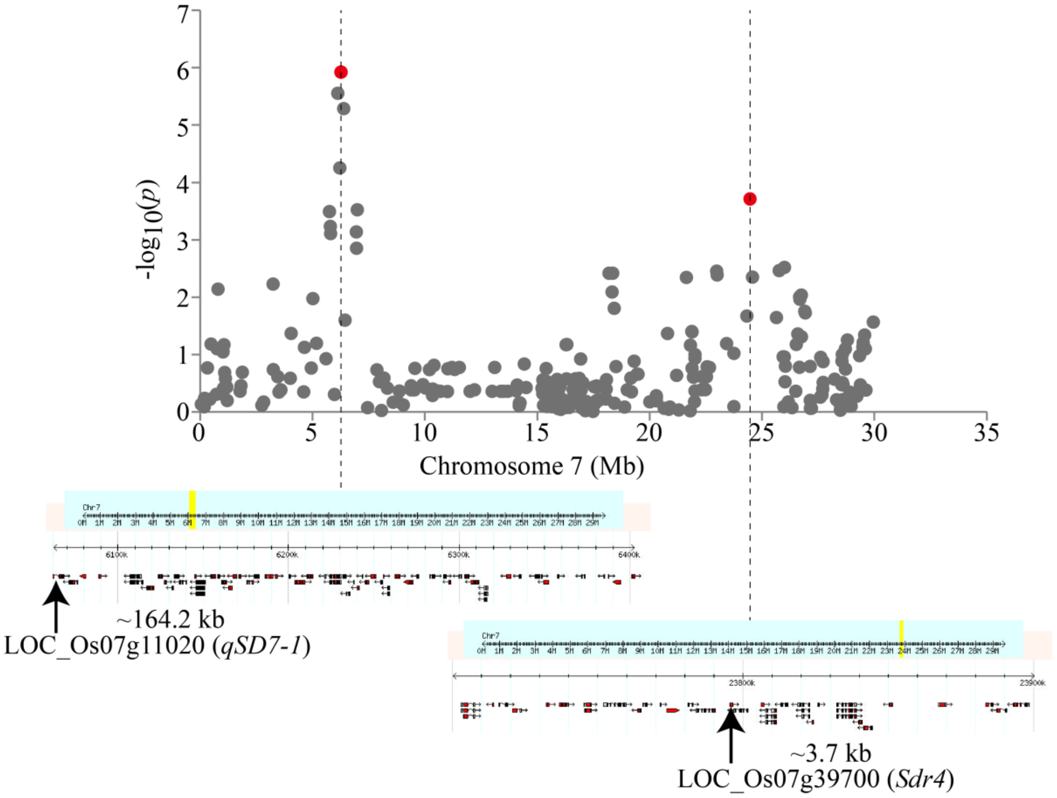

Figure S2 Candidate genes for SNPs associated with seed dormancy on chromosome 7. Red dots represent the lead SNPs. Bottom of the panel shows known gene located within 100 kb adjacent region each side of the lead SNPs.
