# Supplementary material for: Genome-Wide Association Study of Seed Dormancy and the Genomic Consequences of Improvement Footprints in Rice (Oryza sativa L.)
Source: Front Plant Sci. 2018 Jan 5;8:2213. doi: 10.3389/fpls.2017.02213 (PMC5760558; doi:10.3389/fpls.2017.02213)
Supplement: Supplementary file 12 [file Image3.PDF]

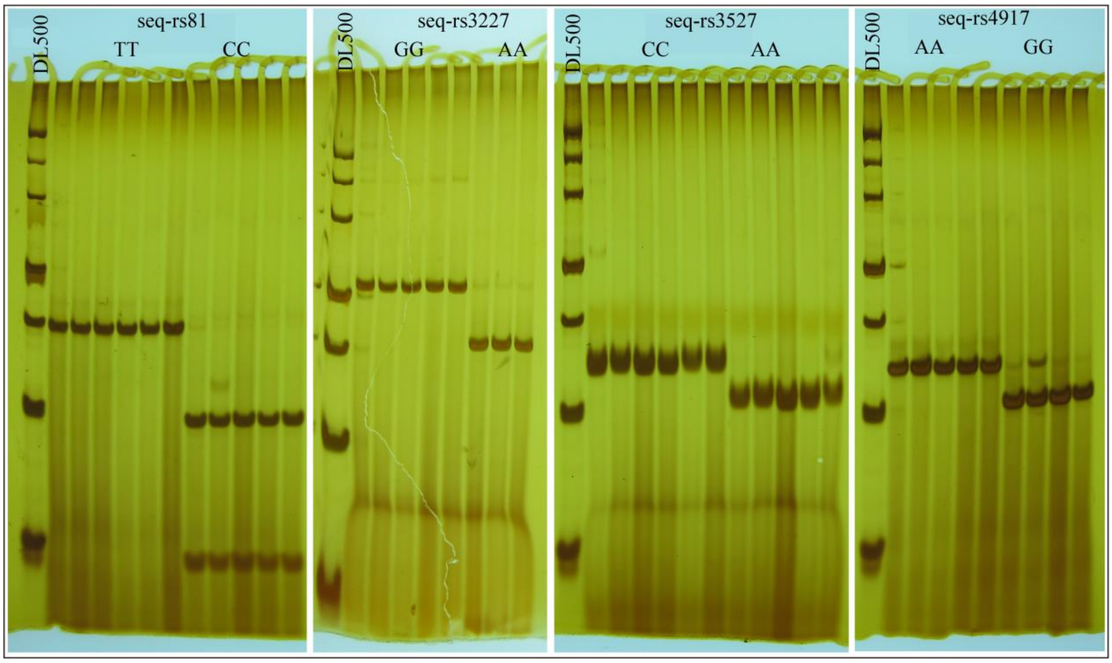

Figure S3 Polyacrylamide gel electrophoresis of dCAPS markers for GWAS lead SNPs. DL500 on the left electrophoresis lane represents DNA marker. The capitals on the top of the polyacrylamide gel electrophoresis represent different alleles of the GWAS lead SNPs.
