# Supplementary material for: Genome-Wide Association Study of Seed Dormancy and the Genomic Consequences of Improvement Footprints in Rice (Oryza sativa L.)
Source: Front Plant Sci. 2018 Jan 5;8:2213. doi: 10.3389/fpls.2017.02213 (PMC5760558; doi:10.3389/fpls.2017.02213)
Supplement: Supplementary file 13 [file Image4.PDF]

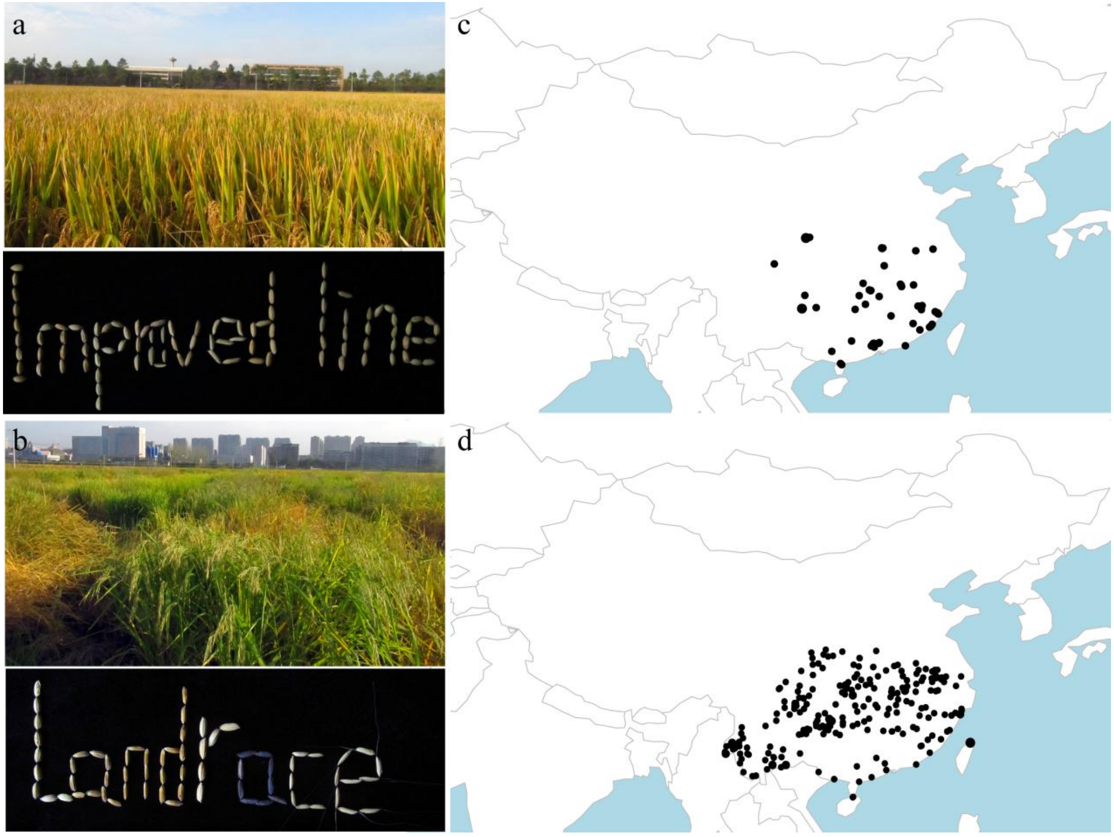

Figure S4 Morphological changes from landrace to improved line during rice improvement. (a-b) Great changes of rice plant architecture and grain traits. (c-d) Geographical distribution of improvement lines and landraces.
