# Supplementary figures and images for: Genome-Wide Association Study of Seed Dormancy and the Genomic Consequences of Improvement Footprints in Rice (Oryza sativa L.)
Source: Front Plant Sci. 2018 Jan 5;8:2213. doi: 10.3389/fpls.2017.02213 (PMC5760558; doi:10.3389/fpls.2017.02213)

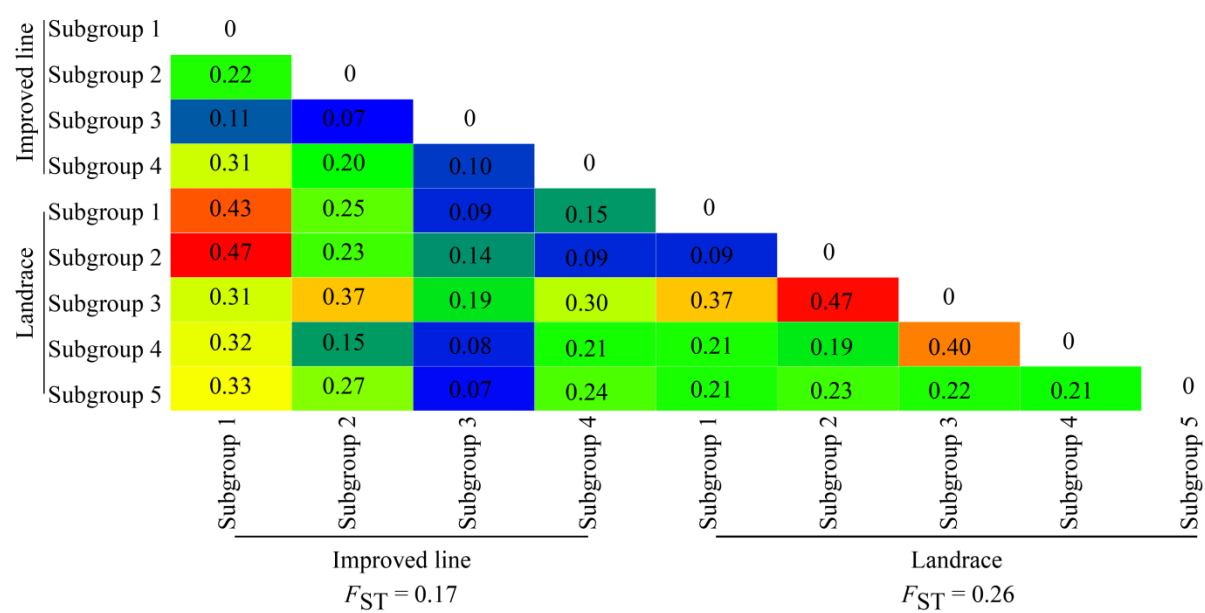

Figure S5 Population differentiation ( $F_{ST}$ ) among different subgroups.

Supplement: Supplementary file 14 [file Image5.PDF]
